# Implementation of a nonprofit specialty clinic for vascular anomalies and birthmarks in Vietnam: A 12-year service evaluation

**DOI:** 10.1016/j.jdin.2026.08.015

**Published:** 2026-09-14

**Authors:** Mitchell Hanson, Allison Chiou, Minh Van Hoang, Vien The Tran, Tri Cao Le, Vy Truc Hoang Nguyen, Anh Quynh Ngoc Phan, Thuy Phung, Stuart Nelson, Martin Mihm, Richard Rox Anderson, Thanh Nga Tran

**Affiliations:** a Department of Pediatrics, Mass General Brigham for Children, Boston, Massachusetts; b University of Minnesota Medical School, Minneapolis, Minnesota; c Medical University Center, Ho Chi Minh, Vietnam; d Vietnam Vascular Anomalies Center, Ho Chi Minh, Vietnam; e University of Texas Health San Antonio, San Antonio, Texas; f Department of Dermatology, Harvard Medical School, Boston, Massachusetts; g Beckman Laser Institute and Medical Clinic, University of California Irvine, Irvine, California; h Brigham and Women’s Hospital, Boston, Massachusetts; i Wellman Center for Photomedicine, Massachusetts General Hospital, Boston, Massachusetts

**Keywords:** birthmark, global dermatology, laser therapy, limited resources setting, non-profit clinic, vascular anomalies

*To the Editor:* Access to specialized care for vascular anomalies and other birthmarks remains limited in many low- and middle-income countries due to shortages of trained clinicians, equipment, and multidisciplinary expertise,^1^^,^^2^ and few reports describe the long-term sustainability of such programs.^3^ The Vietnam Vascular Anomalies Center (VVAC), a nonprofit multidisciplinary referral center established in 2009, expands access to care of vascular anomalies and other birthmarks in Ho Chi Minh City, Vietnam. We performed a retrospective service evaluation to characterize VVAC’s clinical activity and implementation experience over 12 years.

This retrospective review examined clinic records, procedural logs, and administrative databases (2012–2024) from VVAC. Data extracted included examinations, laser procedures, patient age group, historically documented diagnoses, and payment status. Descriptive statistics, χ^2^ tests, linear regression, and Pearson correlation were performed (*P* < .05).

VVAC conducted 63,393 encounters and 27,124 laser procedures (Fig 1), with annual encounters ranging from 1919 to 6389. Pediatric patients accounted for 59,162 (93.3%) examinations and 15,226 (56.1%) laser procedures, while adults accounted for 4231 (6.7%) examinations and 11,898 (43.9%) laser procedures (*P* < .001). While adult services required payment to support financial sustainability, all pediatric examinations and laser procedures were provided free of charge. The most common pediatric laser indications were infantile hemangiomas (37.1%), port-wine stains (18.8%), nevus of Ota (17.1%), and congenital melanocytic nevi (15.1%) (Supplementary Table I, available via Mendeley at https://data.mendeley.com/datasets/3c4ddxx773/5).^4^

**Fig 1:**
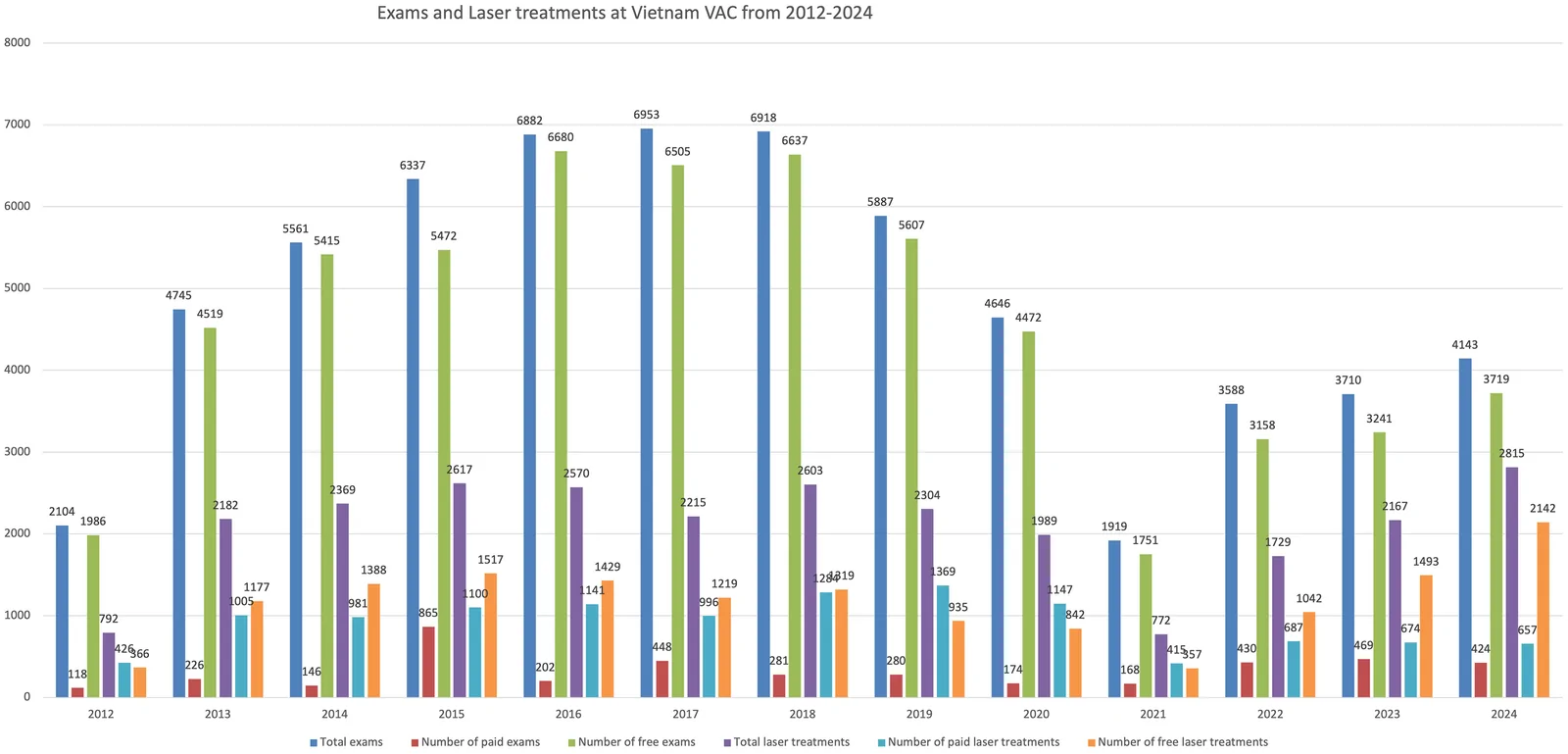
Annual examinations and laser treatments at the Vietnam Vascular Anomalies Center (2012-2024), stratified by paid and free services. Patient volumes fluctuated with growth, pandemic disruption, and recovery.

Implementation approaches and experience-based considerations are summarized in Table I. VVAC developed a multidisciplinary model through local academic partnerships. Its operating model combined philanthropy and cross-subsidization, using adult cosmetic revenue to subsidize free pediatric care. Long-term partnerships with international collaborators and industry supported acquisition and maintenance of laser platforms that would otherwise be difficult to sustain (Supplementary Tables II-IV, available via Mendeley at https://data.mendeley.com/datasets/3c4ddxx773/5, Fig 1).^4^ However, reliance on donated or limited laser platforms, coupled with equipment maintenance requirements and occasional machine downtime, may have influenced treatment selection by necessitating use of available technologies rather than the ideal treatment modality.^5^ Treatment decisions therefore balanced device availability with patient goals, accessibility, and clinical considerations. VVAC supported regional expertise through monthly teleconsultations, annual conferences, hands-on laser training, a train-the-trainer model via in-person, and recorded clinical demonstrations. Limited pediatric propranolol availability, equipment maintenance, and high demand necessitated flexible treatment algorithms (Supplementary Fig 2, available via Mendeley at https://data.mendeley.com/datasets/3c4ddxx773/5), illustrated by cases (Supplementary Cases 1-4, available via Mendeley at https://data.mendeley.com/datasets/3c4ddxx773/5).^4^ Lastly, local stigma and misinformation concerning alternative therapies required targeted counseling and outreach.

**Table I:** Implementation approaches and lessons from 12 years of the Vietnam Vascular Anomalies Center

Domain	Challenge	VVAC approach	Lesson
Sustainable financing	Limited resources and high pediatric demand.	Cross-subsidization through adult cosmetic procedures combined with philanthropy.	Programs may consider diversified funding and cross-subsidization while prospectively monitoring costs, revenue sources, and donor dependence.
Multidisciplinary care	Complex vascular anomalies require multidisciplinary expertise that may not be readily available in resource-limited settings.	Build partnerships with local academic institutions for subspecialty care while gradually expanding in-house expertise over time.	Programs may consider developing local multidisciplinary expertise alongside referral networks as resources expand.
Education and capacity building	Limited local expertise and geographical barriers.	Monthly teleconsultations, annual conferences, annual expert-led live patient demonstrations with recorded case review, laser training courses, and a train-the-trainer model.	Local training may extend the impact of specialty centers, fostering self-sufficiency and reducing long-term reliance on international aid.
Adaptability	Medication shortages, limited access to advanced imaging and non-laser therapies, equipment maintenance, and high patient volume.	Flexible treatment pathways using available therapies and alternative laser platforms to optimize outcomes within patients' logistical and resource constraints, including travel distance, transportation access, and work obligations	Evidence-based care often requires adaptation to local resources and cultural values while recognizing how these constraints may influence treatment selection and diagnosis.
Partnerships	Dependence on donated technology and industry partnerships for acquisition and maintenance advanced laser platforms.	Long-term research and quality improvement collaborations with international faculty and industry partners.	Partnerships may improve access to technology, but programs should continue expanding complementary diagnostic and therapeutic resources as capacity grows, while remaining vigilant that treatment selection is guided by clinical indication rather than by industry-provided equipment availability.
Community engagement	Local stigma and misinformation surrounding vascular anomalies and birthmarks.	Patient and family counseling, educational flyers, community outreach, and public awareness campaigns addressing misinformation and mistrust.	Community engagement should accompany specialty service development to improve awareness, reduce stigma, and increase timely access to care.

*VVAC*, Vietnam Vascular Anomalies Center.

Limitations include the retrospective, encounter-level data, which do not identify unique patients, retention, or longitudinal clinical outcomes and incompletely capture non-laser treatments. Cost, revenue, donor-contribution, and solvency data were unavailable; therefore, financial sustainability and donor dependence could not be evaluated. Diagnostic classification reflected documentation at evaluation, evolving imaging availability, and access to multidisciplinary consultation. These findings describe single-center implementation and sustained service delivery rather than treatment effectiveness or proof of a financially sustainable, generalizable model.

Our experience demonstrates sustained delivery of nonprofit specialty services and offers implementation considerations in multidisciplinary collaboration, clinician education, financing approaches, and adaptation to local resource constraints. Prospective outcome, retention, and financial data are needed before the model’s sustainability and transferability can be established.

## Conflicts of interest

None disclosed.
